# Interplay Between Socioeconomic Markers and Genetic Predisposition to the Time to Dementia Onset

**DOI:** 10.1093/geroni/igaa057.2204

**Published:** 2020-12-16

**Authors:** Olesya Ajnakina, Dorina Cadar, Andrew Steptoe

**Affiliations:** University College London, London, England, United Kingdom

## Abstract

Identifying interplay between socio-economic markers (education and financial resources) and genetic factors influencing time of dementia and Alzheimer’s disease (AD) diagnosis is of central relevance for the development of preventative strategies. Using 7039 individuals aged >50 years from the English Longitudinal Study of Ageing, 320 (4.6%) of whom developed dementia over the 10-year follow-up, we investigated interactions between polygenic score for AD (AD-PGS) and socioeconomic markers on the timing of dementia or AD diagnosis. One standard deviation increase in AD-PGS was associated with an accelerated time to dementia onset by 4.8 months. Interactions between AD-PGS and lower wealth accelerated time to AD diagnosis by 24 months; an interaction between AD-PGS and years of schooling in decelerating time to AD by 3.0 months suggests education serves as protective mechanisms against AD diagnosis. Socio-economic markers are important factors influencing time to dementia and AD, particularly in those with polygenetic predisposition to AD.

